# Supplementary material for: Hot spot prediction in protein-protein interactions by an ensemble system
Source: BMC Syst Biol. 2018 Dec 31;12(Suppl 9):132. doi: 10.1186/s12918-018-0665-8 (PMC6311905; doi:10.1186/s12918-018-0665-8)
Supplement: Supplementary file 2 — All relevant MATLAB code. A simple Matlab implement of our predictor. (ZIP 19,742 kb) [file 12918_2018_665_MOESM2_ESM.zip › Additional file 2/Read ASA/ASA Property/SKEMPI/3BP8A.txt]

 NetSurfP - Protein Surface Accessibility and Secondary Structure Predictions

  

|  |  |  |
| --- | --- | --- |
|  |  | NetSurfP - Protein Surface Accessibility and Secondary Structure Predictions  Technical University of Denmark |

  


---

```
# For publication of results, please cite:
# A generic method for assignment of reliability scores applied to solvent accessibility predictions.
# Bent Petersen, Thomas Nordahl Petersen, Pernille Andersen, Morten Nielsen and Claus Lundegaard
# BMC Structural Biology 2009, 9:51 doi:10.1186/1472-6807-9-51
#
# Column 1: Class assignment - B for buried or E for Exposed - Threshold: 25% exposure, but not based on RSA
# Column 2: Amino acid
# Column 3: Sequence name
# Column 4: Amino acid number
# Column 5: Relative Surface Accessibility - RSA
# Column 6: Absolute Surface Accessibility
# Column 7: Z-fit score for RSA prediction
# Column 8: Probability for Alpha-Helix
# Column 9: Probability for Beta-strand
# Column 10: Probability for Coil
E D  3BP8A                  1    0.874 125.943   0.481   0.016   0.005   0.979
E Q  3BP8A                  2    0.570 101.748   1.035   0.782   0.003   0.216
B I  3BP8A                  3    0.174  32.116  -0.488   0.858   0.002   0.139
E K  3BP8A                  4    0.358  73.682   1.685   0.879   0.010   0.111
E Q  3BP8A                  5    0.638 113.983   1.200   0.879   0.010   0.111
E T  3BP8A                  6    0.373  51.804   1.588   0.858   0.002   0.139
B N  3BP8A                  7    0.102  14.904  -0.361   0.782   0.003   0.216
E A  3BP8A                  8    0.309  34.074   0.352   0.970   0.001   0.030
E G  3BP8A                  9    0.411  32.330   1.270   0.970   0.001   0.030
E A  3BP8A                 10    0.264  29.049   2.251   0.970   0.001   0.030
B V  3BP8A                 11    0.022   3.351   1.234   0.970   0.001   0.030
B Y  3BP8A                 12    0.069  14.852   0.746   0.970   0.001   0.030
E R  3BP8A                 13    0.441 100.966   2.092   0.988   0.000   0.012
B L  3BP8A                 14    0.140  25.689   1.884   0.988   0.000   0.012
B I  3BP8A                 15    0.029   5.439   0.781   0.970   0.001   0.030
E D  3BP8A                 16    0.307  44.239   1.264   0.923   0.002   0.076
E Q  3BP8A                 17    0.677 120.841   1.183   0.782   0.003   0.216
E L  3BP8A                 18    0.534  97.849   0.308   0.246   0.004   0.750
E G  3BP8A                 19    0.471  37.052  -0.536   0.016   0.005   0.979
E P  3BP8A                 20    0.543  77.123  -0.356   0.016   0.005   0.979
B V  3BP8A                 21    0.142  21.902  -0.501   0.018   0.047   0.935
B S  3BP8A                 22    0.240  28.128  -0.001   0.053   0.043   0.903
B R  3BP8A                 23    0.235  53.861   0.651   0.938   0.007   0.055
E I  3BP8A                 24    0.346  63.936   0.274   0.923   0.002   0.076
E D  3BP8A                 25    0.464  66.934   1.435   0.970   0.001   0.030
B L  3BP8A                 26    0.052   9.576   0.602   0.988   0.000   0.012
B S  3BP8A                 27    0.081   9.481  -0.509   0.970   0.001   0.030
E R  3BP8A                 28    0.684 156.590   1.415   0.923   0.002   0.076
E L  3BP8A                 29    0.493  90.177   0.957   0.858   0.002   0.139
B A  3BP8A                 30    0.144  15.924   0.735   0.502   0.002   0.495
E Q  3BP8A                 31    0.731 130.610  -0.134   0.053   0.005   0.942
B L  3BP8A                 32    0.135  24.792  -0.032   0.016   0.005   0.979
E A  3BP8A                 33    0.406  44.763  -0.213   0.005   0.015   0.979
E P  3BP8A                 34    0.463  65.728   0.467   0.858   0.002   0.139
E A  3BP8A                 35    0.453  49.899  -0.785   0.938   0.007   0.055
B S  3BP8A                 36    0.217  25.444   0.633   0.938   0.007   0.055
B I  3BP8A                 37    0.053   9.861  -0.311   0.970   0.001   0.030
B T  3BP8A                 38    0.218  30.264   0.244   0.970   0.001   0.030
E K  3BP8A                 39    0.471  96.823   1.629   0.970   0.001   0.030
B I  3BP8A                 40    0.098  18.130   1.057   0.970   0.001   0.030
B V  3BP8A                 41    0.049   7.516   0.963   0.988   0.000   0.012
E R  3BP8A                 42    0.459 105.225   1.676   0.988   0.000   0.012
E E  3BP8A                 43    0.423  73.811   2.353   0.970   0.001   0.030
B M  3BP8A                 44    0.041   8.144   0.504   0.970   0.001   0.030
E L  3BP8A                 45    0.257  46.983   1.133   0.923   0.002   0.076
E E  3BP8A                 46    0.774 135.218   1.174   0.858   0.002   0.139
E A  3BP8A                 47    0.456  50.273   1.963   0.246   0.004   0.750
E H  3BP8A                 48    0.508  92.387   0.363   0.018   0.019   0.964
B L  3BP8A                 49    0.152  27.831   1.527   0.056   0.142   0.802
B V  3BP8A                 50    0.070  10.836   0.034   0.074   0.484   0.442
E Q  3BP8A                 51    0.466  83.299   1.319   0.079   0.592   0.329
E E  3BP8A                 52    0.431  75.278   0.891   0.074   0.484   0.442
E L  3BP8A                 53    0.450  82.450   0.437   0.019   0.141   0.840
E E  3BP8A                 54    0.608 106.218  -0.981   0.018   0.088   0.893
E V  3BP8A                 55    0.360  55.393  -0.110   0.021   0.279   0.699
E G  3BP8A                 56    0.279  21.942  -0.828   0.022   0.552   0.426
B L  3BP8A                 57    0.099  18.127   0.341   0.021   0.756   0.223
E V  3BP8A                 58    0.418  64.262   0.114   0.018   0.846   0.136
B V  3BP8A                 59    0.095  14.540   0.379   0.021   0.756   0.223
B E  3BP8A                 60    0.114  19.916   0.244   0.005   0.262   0.733
E T  3BP8A                 61    0.696  96.494  -0.751   0.005   0.015   0.979
E E  3BP8A                 62    0.697 121.696  -0.119   0.003   0.003   0.994
B A  3BP8A                 63    0.168  18.503   0.600   0.004   0.085   0.910
B W  3BP8A                 64    0.116  27.802   0.533   0.004   0.420   0.576
B H  3BP8A                 65    0.097  17.553   1.366   0.001   0.900   0.099
B Y  3BP8A                 66    0.026   5.663   0.758   0.001   0.959   0.040
B L  3BP8A                 67    0.020   3.589   1.408   0.000   0.983   0.017
B S  3BP8A                 68    0.020   2.332   0.780   0.000   0.983   0.017
B L  3BP8A                 69    0.039   7.123   0.517   0.000   0.983   0.017
B R  3BP8A                 70    0.140  32.129   0.509   0.000   0.983   0.017
B I  3BP8A                 71    0.071  13.227   0.664   0.002   0.816   0.182
B S  3BP8A                 72    0.243  28.480   0.138   0.004   0.197   0.799
E R  3BP8A                 73    0.395  90.363  -0.523   0.018   0.019   0.964
E G  3BP8A                 74    0.450  35.399  -0.445   0.019   0.141   0.840
E E  3BP8A                 75    0.276  48.200   1.555   0.002   0.816   0.182
B I  3BP8A                 76    0.039   7.178   0.597   0.001   0.959   0.040
B F  3BP8A                 77    0.171  34.360   1.561   0.001   0.959   0.040
B L  3BP8A                 78    0.026   4.761   0.803   0.000   0.983   0.017
B A  3BP8A                 79    0.025   2.766   0.976   0.000   0.983   0.017
B L  3BP8A                 80    0.034   6.317   0.289   0.000   0.983   0.017
B R  3BP8A                 81    0.046  10.465   0.578   0.000   0.983   0.017
B D  3BP8A                 82    0.116  16.759   0.360   0.003   0.718   0.279
E L  3BP8A                 83    0.377  68.955  -0.741   0.004   0.085   0.910
E S  3BP8A                 84    0.671  78.629   0.668   0.005   0.015   0.979
B S  3BP8A                 85    0.240  28.093  -0.204   0.005   0.045   0.951
E K  3BP8A                 86    0.514 105.648   1.034   0.004   0.514   0.481
E L  3BP8A                 87    0.277  50.645   1.173   0.001   0.900   0.099
E V  3BP8A                 88    0.239  36.734   1.141   0.006   0.962   0.032
E V  3BP8A                 89    0.389  59.851   0.270   0.001   0.959   0.040
E E  3BP8A                 90    0.454  79.279   1.120   0.001   0.959   0.040
E E  3BP8A                 91    0.377  65.844   0.683   0.001   0.959   0.040
E S  3BP8A                 92    0.606  71.070   0.746   0.001   0.959   0.040
B Q  3BP8A                 93    0.180  32.094   1.722   0.001   0.900   0.099
E E  3BP8A                 94    0.530  92.504   0.363   0.004   0.616   0.381
B L  3BP8A                 95    0.129  23.693   0.099   0.004   0.138   0.858
E A  3BP8A                 96    0.611  67.277   0.259   0.005   0.045   0.951
E L  3BP8A                 97    0.470  86.130  -0.638   0.018   0.019   0.964
E K  3BP8A                 98    0.716 147.343  -0.620   0.016   0.005   0.979
E D  3BP8A                 99    0.543  78.318   0.301   0.016   0.005   0.979
B D  3BP8A                100    0.114  16.499  -0.447   0.858   0.002   0.139
E S  3BP8A                101    0.675  79.098   1.066   0.970   0.001   0.030
E P  3BP8A                102    0.498  70.709   1.861   0.970   0.001   0.030
B L  3BP8A                103    0.041   7.434   0.476   0.970   0.001   0.030
B L  3BP8A                104    0.043   7.910   0.762   0.970   0.001   0.030
E D  3BP8A                105    0.538  77.468   1.945   0.970   0.001   0.030
E R  3BP8A                106    0.283  64.761   2.142   0.970   0.001   0.030
B I  3BP8A                107    0.016   3.053   1.548   0.988   0.000   0.012
B I  3BP8A                108    0.137  25.327   1.276   0.970   0.001   0.030
E S  3BP8A                109    0.557  65.316   1.921   0.970   0.001   0.030
B H  3BP8A                110    0.106  19.227   1.321   0.970   0.001   0.030
B I  3BP8A                111    0.019   3.441   1.333   0.970   0.001   0.030
E D  3BP8A                112    0.388  55.868   2.361   0.970   0.001   0.030
E Q  3BP8A                113    0.503  89.907   2.124   0.970   0.001   0.030
B F  3BP8A                114    0.045   8.991   0.576   0.970   0.001   0.030
B F  3BP8A                115    0.119  23.783   0.292   0.970   0.001   0.030
E I  3BP8A                116    0.697 128.927   1.369   0.923   0.002   0.076
E R  3BP8A                117    0.584 133.667   1.243   0.858   0.002   0.139
B H  3BP8A                118    0.125  22.756   0.464   0.502   0.002   0.495
E Q  3BP8A                119    0.728 130.110  -0.035   0.109   0.005   0.886
E K  3BP8A                120    0.319  65.659  -0.047   0.053   0.005   0.942
E K  3BP8A                121    0.608 125.066   0.097   0.053   0.005   0.942
E L  3BP8A                122    0.467  85.581  -0.719   0.058   0.017   0.925
E E  3BP8A                123    0.485  84.817  -0.599   0.018   0.019   0.964
E R  3BP8A                124    0.433  99.043   1.151   0.018   0.047   0.935
B L  3BP8A                125    0.028   5.090   0.102   0.004   0.514   0.481
B T  3BP8A                126    0.179  24.841   0.580   0.003   0.718   0.279
B S  3BP8A                127    0.033   3.914  -0.842   0.001   0.900   0.099
B I  3BP8A                128    0.020   3.737   1.126   0.001   0.959   0.040
B A  3BP8A                129    0.021   2.281   0.306   0.001   0.959   0.040
B I  3BP8A                130    0.027   5.088   0.432   0.001   0.959   0.040
B T  3BP8A                131    0.026   3.551  -0.295   0.001   0.900   0.099
B L  3BP8A                132    0.043   7.800   0.228   0.004   0.514   0.481
B P  3BP8A                133    0.089  12.615  -0.485   0.005   0.336   0.660
B G  3BP8A                134    0.063   4.942  -0.797   0.005   0.336   0.660
B I  3BP8A                135    0.239  44.123   0.157   0.004   0.514   0.481
B I  3BP8A                136    0.084  15.540  -0.053   0.004   0.514   0.481
B D  3BP8A                137    0.213  30.737   0.610   0.021   0.279   0.699
E T  3BP8A                138    0.459  63.622  -0.062   0.018   0.047   0.935
E E  3BP8A                139    0.754 131.811   0.103   0.018   0.047   0.935
E N  3BP8A                140    0.461  67.490   0.490   0.018   0.047   0.935
B G  3BP8A                141    0.190  14.929  -0.832   0.004   0.138   0.858
B I  3BP8A                142    0.218  40.293   1.356   0.002   0.816   0.182
B V  3BP8A                143    0.038   5.841  -0.949   0.001   0.900   0.099
B H  3BP8A                144    0.215  39.090   0.332   0.018   0.846   0.136
B R  3BP8A                145    0.287  65.654  -0.727   0.004   0.514   0.481
B M  3BP8A                146    0.051  10.225   0.258   0.019   0.141   0.840
E P  3BP8A                147    0.378  53.681  -1.193   0.018   0.047   0.935
E F  3BP8A                148    0.349  69.984  -0.897   0.018   0.047   0.935
B Y  3BP8A                149    0.084  18.015   0.285   0.058   0.017   0.925
E E  3BP8A                150    0.546  95.474   0.026   0.018   0.019   0.964
E D  3BP8A                151    0.413  59.571  -0.511   0.018   0.019   0.964
B V  3BP8A                152    0.121  18.598  -0.299   0.058   0.017   0.925
E K  3BP8A                153    0.634 130.414   0.189   0.018   0.019   0.964
E E  3BP8A                154    0.777 135.812  -0.118   0.005   0.015   0.979
B M  3BP8A                155    0.144  28.794   1.913   0.018   0.019   0.964
E P  3BP8A                156    0.557  79.010   1.476   0.181   0.016   0.803
B L  3BP8A                157    0.038   6.921   0.436   0.782   0.003   0.216
B G  3BP8A                158    0.216  16.983   1.357   0.923   0.002   0.076
E E  3BP8A                159    0.613 107.021   1.684   0.970   0.001   0.030
E A  3BP8A                160    0.279  30.702   1.892   0.970   0.001   0.030
B L  3BP8A                161    0.022   3.955   1.132   0.970   0.001   0.030
E E  3BP8A                162    0.443  77.427   1.910   0.923   0.002   0.076
E Q  3BP8A                163    0.793 141.558   1.575   0.858   0.002   0.139
E H  3BP8A                164    0.390  70.977   1.027   0.782   0.003   0.216
B T  3BP8A                165    0.087  12.095   0.759   0.321   0.003   0.675
E G  3BP8A                166    0.828  65.195   1.036   0.003   0.003   0.994
B V  3BP8A                167    0.099  15.216   1.523   0.005   0.015   0.979
E P  3BP8A                168    0.299  42.471   1.343   0.005   0.262   0.733
B V  3BP8A                169    0.021   3.274   1.350   0.001   0.959   0.040
B Y  3BP8A                170    0.114  24.405   1.462   0.000   0.983   0.017
B I  3BP8A                171    0.020   3.737   1.056   0.000   0.983   0.017
B Q  3BP8A                172    0.075  13.484   0.323   0.011   0.918   0.071
B H  3BP8A                173    0.080  14.570  -0.707   0.066   0.296   0.638
B D  3BP8A                174    0.100  14.381  -0.164   0.430   0.016   0.555
B I  3BP8A                175    0.028   5.143   0.107   0.622   0.015   0.363
B S  3BP8A                176    0.091  10.630   0.291   0.802   0.014   0.185
B A  3BP8A                177    0.017   1.829   1.316   0.911   0.033   0.057
B W  3BP8A                178    0.027   6.397   0.635   0.938   0.007   0.055
B T  3BP8A                179    0.024   3.357   0.447   0.938   0.007   0.055
B M  3BP8A                180    0.048   9.585   0.626   0.938   0.007   0.055
B A  3BP8A                181    0.026   2.810   0.172   0.975   0.003   0.022
B E  3BP8A                182    0.042   7.285   0.027   0.970   0.001   0.030
B A  3BP8A                183    0.126  13.863   0.841   0.970   0.001   0.030
B L  3BP8A                184    0.161  29.406   0.837   0.923   0.002   0.076
B F  3BP8A                185    0.228  45.659   0.551   0.858   0.002   0.139
B G  3BP8A                186    0.234  18.416  -2.083   0.502   0.002   0.495
E A  3BP8A                187    0.419  46.119  -0.501   0.176   0.004   0.820
E S  3BP8A                188    0.380  44.559  -1.533   0.115   0.016   0.868
E R  3BP8A                189    0.483 110.676   0.347   0.058   0.017   0.925
E G  3BP8A                190    0.658  51.816  -0.367   0.018   0.019   0.964
B A  3BP8A                191    0.122  13.466   0.679   0.018   0.019   0.964
E R  3BP8A                192    0.529 121.072   0.948   0.005   0.015   0.979
B D  3BP8A                193    0.062   8.905  -0.251   0.005   0.336   0.660
B V  3BP8A                194    0.020   3.135   1.205   0.001   0.900   0.099
B I  3BP8A                195    0.023   4.329   0.813   0.001   0.959   0.040
B Q  3BP8A                196    0.033   5.840   0.460   0.000   0.983   0.017
B V  3BP8A                197    0.022   3.381   1.361   0.000   0.983   0.017
B V  3BP8A                198    0.080  12.281   0.629   0.001   0.959   0.040
B I  3BP8A                199    0.120  22.200  -0.061   0.003   0.718   0.279
B D  3BP8A                200    0.190  27.393  -1.132   0.019   0.141   0.840
B H  3BP8A                201    0.299  54.370  -0.677   0.053   0.043   0.903
B N  3BP8A                202    0.126  18.476  -2.170   0.052   0.084   0.864
B V  3BP8A                203    0.110  16.938   0.210   0.056   0.142   0.802
B G  3BP8A                204    0.065   5.076  -2.228   0.022   0.359   0.619
B A  3BP8A                205    0.046   5.058  -0.824   0.023   0.655   0.322
B G  3BP8A                206    0.028   2.196  -0.245   0.011   0.918   0.071
B V  3BP8A                207    0.063   9.668   0.708   0.006   0.962   0.032
B I  3BP8A                208    0.038   7.049   0.297   0.006   0.962   0.032
B T  3BP8A                209    0.099  13.801   0.318   0.002   0.816   0.182
E D  3BP8A                210    0.428  61.675  -0.356   0.004   0.138   0.858
E G  3BP8A                211    0.372  29.253  -0.782   0.005   0.015   0.979
E H  3BP8A                212    0.330  60.118   0.469   0.023   0.655   0.322
B L  3BP8A                213    0.095  17.468  -0.132   0.021   0.756   0.223
B L  3BP8A                214    0.126  23.126   0.161   0.023   0.655   0.322
E H  3BP8A                215    0.359  65.320   0.637   0.022   0.359   0.619
B A  3BP8A                216    0.087   9.609  -2.309   0.019   0.141   0.840
E G  3BP8A                217    0.514  40.428  -1.380   0.005   0.015   0.979
E S  3BP8A                218    0.402  47.091  -1.406   0.005   0.015   0.979
B S  3BP8A                219    0.237  27.812  -1.636   0.018   0.019   0.964
B S  3BP8A                220    0.142  16.642  -1.048   0.018   0.047   0.935
B L  3BP8A                221    0.036   6.573  -1.334   0.020   0.205   0.775
B V  3BP8A                222    0.045   6.978  -1.176   0.005   0.336   0.660
B E  3BP8A                223    0.035   6.114  -1.033   0.004   0.616   0.381
B I  3BP8A                224    0.030   5.476  -0.282   0.002   0.816   0.182
B G  3BP8A                225    0.026   2.030  -0.634   0.003   0.718   0.279
B H  3BP8A                226    0.064  11.551  -0.343   0.004   0.616   0.381
B T  3BP8A                227    0.029   4.036   0.589   0.002   0.816   0.182
B Q  3BP8A                228    0.130  23.254   0.879   0.021   0.756   0.223
B V  3BP8A                229    0.055   8.500   0.315   0.023   0.655   0.322
E D  3BP8A                230    0.287  41.299   0.677   0.005   0.262   0.733
E P  3BP8A                231    0.498  70.695  -1.153   0.004   0.085   0.910
E Y  3BP8A                232    0.766 163.716  -2.286   0.018   0.019   0.964
E G  3BP8A                233    0.437  34.400  -1.749   0.018   0.019   0.964
E K  3BP8A                234    0.585 120.252  -0.965   0.018   0.047   0.935
E R  3BP8A                235    0.533 121.943   0.099   0.018   0.088   0.893
B C  3BP8A                236    0.103  14.517  -1.364   0.018   0.088   0.893
E Y  3BP8A                237    0.458  97.768  -0.942   0.018   0.047   0.935
B C  3BP8A                238    0.102  14.307  -1.670   0.018   0.019   0.964
E G  3BP8A                239    0.315  24.767  -1.633   0.018   0.019   0.964
E N  3BP8A                240    0.398  58.282  -1.134   0.018   0.047   0.935
E H  3BP8A                241    0.370  67.230   0.649   0.052   0.084   0.864
B G  3BP8A                242    0.096   7.571  -0.752   0.113   0.087   0.800
B C  3BP8A                243    0.070   9.828   0.490   0.307   0.165   0.527
B L  3BP8A                244    0.037   6.756  -0.124   0.538   0.173   0.289
B E  3BP8A                245    0.065  11.408  -0.084   0.605   0.105   0.290
B T  3BP8A                246    0.149  20.652  -0.023   0.605   0.105   0.290
B I  3BP8A                247    0.080  14.874   0.615   0.538   0.173   0.289
B A  3BP8A                248    0.030   3.295  -0.172   0.216   0.235   0.548
B S  3BP8A                249    0.050   5.801  -0.556   0.191   0.086   0.723
B V  3BP8A                250    0.114  17.568  -0.891   0.879   0.010   0.111
E D  3BP8A                251    0.335  48.346   1.035   0.970   0.001   0.030
B S  3BP8A                252    0.087  10.150  -0.366   0.970   0.001   0.030
B I  3BP8A                253    0.026   4.828   0.415   0.988   0.000   0.012
B L  3BP8A                254    0.103  18.951   0.601   0.988   0.000   0.012
E E  3BP8A                255    0.386  67.382   1.177   0.970   0.001   0.030
B L  3BP8A                256    0.212  38.836   1.500   0.970   0.001   0.030
B A  3BP8A                257    0.039   4.276  -0.305   0.970   0.001   0.030
E Q  3BP8A                258    0.318  56.831   0.707   0.970   0.001   0.030
E L  3BP8A                259    0.605 110.867   0.978   0.923   0.002   0.076
E R  3BP8A                260    0.281  64.441   0.598   0.858   0.002   0.139
B L  3BP8A                261    0.167  30.559  -0.705   0.694   0.003   0.303
E N  3BP8A                262    0.628  91.998  -0.338   0.406   0.004   0.590
E Q  3BP8A                263    0.711 127.002   0.118   0.321   0.003   0.675
E S  3BP8A                264    0.407  47.700  -1.005   0.176   0.004   0.820
E M  3BP8A                265    0.471  94.307  -1.265   0.109   0.005   0.886
E H  3BP8A                266    0.601 109.340  -0.072   0.053   0.005   0.942
E G  3BP8A                267    0.515  40.507  -1.205   0.053   0.005   0.942
E Q  3BP8A                268    0.675 120.591  -0.429   0.018   0.019   0.964
E P  3BP8A                269    0.608  86.204  -0.136   0.018   0.019   0.964
B L  3BP8A                270    0.097  17.761  -0.127   0.005   0.045   0.951
E T  3BP8A                271    0.409  56.784   1.456   0.018   0.019   0.964
B V  3BP8A                272    0.051   7.823   0.103   0.923   0.002   0.076
E D  3BP8A                273    0.509  73.390   1.813   0.970   0.001   0.030
E S  3BP8A                274    0.360  42.180   1.919   0.970   0.001   0.030
B L  3BP8A                275    0.026   4.779   1.031   0.970   0.001   0.030
B C  3BP8A                276    0.087  12.173   1.077   0.970   0.001   0.030
E Q  3BP8A                277    0.526  93.890   1.979   0.970   0.001   0.030
B A  3BP8A                278    0.145  15.957   1.197   0.923   0.002   0.076
B A  3BP8A                279    0.058   6.414   0.409   0.923   0.002   0.076
E L  3BP8A                280    0.617 112.936   1.581   0.782   0.003   0.216
E R  3BP8A                281    0.704 161.170   1.368   0.246   0.004   0.750
E G  3BP8A                282    0.604  47.543  -0.695   0.016   0.005   0.979
E D  3BP8A                283    0.248  35.794   0.530   0.003   0.003   0.994
E L  3BP8A                284    0.653 119.601   1.608   0.858   0.002   0.139
E L  3BP8A                285    0.361  66.081   1.857   0.938   0.007   0.055
B A  3BP8A                286    0.021   2.281   1.170   0.975   0.003   0.022
E K  3BP8A                287    0.259  53.256   2.060   0.975   0.003   0.022
E D  3BP8A                288    0.507  73.102   1.965   0.970   0.001   0.030
B I  3BP8A                289    0.069  12.802   1.441   0.988   0.000   0.012
B I  3BP8A                290    0.027   4.976   1.320   0.970   0.001   0.030
E T  3BP8A                291    0.396  54.897   1.937   0.970   0.001   0.030
E G  3BP8A                292    0.382  30.032   2.015   0.970   0.001   0.030
B V  3BP8A                293    0.058   8.930   1.218   0.970   0.001   0.030
B G  3BP8A                294    0.038   3.022   0.277   0.970   0.001   0.030
E A  3BP8A                295    0.390  43.011   1.075   0.970   0.001   0.030
B H  3BP8A                296    0.190  34.634   1.149   0.970   0.001   0.030
B V  3BP8A                297    0.053   8.100   0.890   0.988   0.000   0.012
B G  3BP8A                298    0.051   3.990  -0.783   0.988   0.000   0.012
B R  3BP8A                299    0.216  49.487   1.189   0.988   0.000   0.012
B I  3BP8A                300    0.053   9.731   0.524   0.988   0.000   0.012
B L  3BP8A                301    0.039   7.104   0.171   0.988   0.000   0.012
B A  3BP8A                302    0.114  12.563   0.176   0.988   0.000   0.012
B I  3BP8A                303    0.105  19.444  -0.056   0.988   0.000   0.012
B M  3BP8A                304    0.034   6.763   0.394   0.970   0.001   0.030
B V  3BP8A                305    0.064   9.821   0.397   0.970   0.001   0.030
B N  3BP8A                306    0.146  21.418   0.282   0.938   0.007   0.055
B L  3BP8A                307    0.049   9.045  -0.047   0.879   0.010   0.111
B F  3BP8A                308    0.067  13.427   0.206   0.622   0.015   0.363
B N  3BP8A                309    0.245  35.853   0.391   0.053   0.005   0.942
B P  3BP8A                310    0.087  12.289   0.410   0.003   0.003   0.994
E Q  3BP8A                311    0.476  84.978   0.759   0.005   0.015   0.979
B K  3BP8A                312    0.095  19.480   1.183   0.003   0.718   0.279
B I  3BP8A                313    0.016   2.886   1.787   0.001   0.959   0.040
B L  3BP8A                314    0.030   5.438   0.667   0.000   0.983   0.017
B I  3BP8A                315    0.022   4.144   0.697   0.001   0.959   0.040
B G  3BP8A                316    0.067   5.304  -1.249   0.004   0.514   0.481
B S  3BP8A                317    0.106  12.376  -1.460   0.018   0.088   0.893
B P  3BP8A                318    0.268  38.029  -1.199   0.184   0.043   0.773
B L  3BP8A                319    0.143  26.110  -0.520   0.339   0.016   0.645
B S  3BP8A                320    0.125  14.673  -0.991   0.257   0.016   0.727
E K  3BP8A                321    0.474  97.461  -0.460   0.176   0.004   0.820
B A  3BP8A                322    0.201  22.172  -0.863   0.246   0.004   0.750
B A  3BP8A                323    0.262  28.916   0.037   0.406   0.004   0.590
E D  3BP8A                324    0.701 100.956   0.097   0.782   0.003   0.216
E I  3BP8A                325    0.370  68.432   0.776   0.858   0.002   0.139
B L  3BP8A                326    0.037   6.775  -0.348   0.923   0.002   0.076
B F  3BP8A                327    0.135  27.034   1.124   0.970   0.001   0.030
E P  3BP8A                328    0.613  86.928   1.587   0.970   0.001   0.030
E V  3BP8A                329    0.293  45.034   1.986   0.970   0.001   0.030
B I  3BP8A                330    0.013   2.423   1.636   0.970   0.001   0.030
E S  3BP8A                331    0.308  36.062   2.108   0.988   0.000   0.012
E D  3BP8A                332    0.623  89.832   1.911   0.988   0.000   0.012
B S  3BP8A                333    0.201  23.534   1.554   0.988   0.000   0.012
B I  3BP8A                334    0.022   4.051   1.138   0.970   0.001   0.030
E R  3BP8A                335    0.434  99.478   1.670   0.970   0.001   0.030
E Q  3BP8A                336    0.659 117.644   1.129   0.858   0.002   0.139
E Q  3BP8A                337    0.269  47.990   0.863   0.782   0.003   0.216
B A  3BP8A                338    0.137  15.141  -1.092   0.502   0.002   0.495
B L  3BP8A                339    0.277  50.719   0.293   0.321   0.003   0.675
E P  3BP8A                340    0.526  74.682  -0.663   0.181   0.016   0.803
E A  3BP8A                341    0.507  55.893  -1.440   0.115   0.016   0.868
E Y  3BP8A                342    0.364  77.701  -1.422   0.058   0.017   0.925
E S  3BP8A                343    0.319  37.422  -0.868   0.058   0.017   0.925
E Q  3BP8A                344    0.623 111.179  -0.237   0.018   0.019   0.964
E H  3BP8A                345    0.578 105.084   0.285   0.005   0.015   0.979
B I  3BP8A                346    0.039   7.215   0.061   0.004   0.138   0.858
E S  3BP8A                347    0.413  48.427   1.922   0.002   0.816   0.182
B V  3BP8A                348    0.038   5.871  -0.394   0.001   0.959   0.040
E E  3BP8A                349    0.295  51.571   1.422   0.001   0.959   0.040
E S  3BP8A                350    0.322  37.692   0.029   0.002   0.816   0.182
B T  3BP8A                351    0.074  10.236  -1.097   0.005   0.336   0.660
E Q  3BP8A                352    0.589 105.195   0.473   0.020   0.205   0.775
B F  3BP8A                353    0.216  43.452  -0.264   0.019   0.141   0.840
E S  3BP8A                354    0.421  49.294  -0.733   0.018   0.047   0.935
E N  3BP8A                355    0.577  84.400  -1.431   0.109   0.005   0.886
B Q  3BP8A                356    0.281  50.133  -0.443   0.246   0.004   0.750
B G  3BP8A                357    0.036   2.794  -0.686   0.694   0.003   0.303
B T  3BP8A                358    0.083  11.443  -0.060   0.879   0.010   0.111
B M  3BP8A                359    0.023   4.702   0.658   0.938   0.007   0.055
B A  3BP8A                360    0.066   7.306   0.797   0.938   0.007   0.055
B G  3BP8A                361    0.027   2.117  -0.208   0.975   0.003   0.022
B A  3BP8A                362    0.021   2.347   0.835   0.975   0.003   0.022
B A  3BP8A                363    0.032   3.581  -0.500   0.970   0.001   0.030
B L  3BP8A                364    0.097  17.724   1.317   0.988   0.000   0.012
B V  3BP8A                365    0.051   7.808   1.134   0.988   0.000   0.012
B K  3BP8A                366    0.049   9.997   0.588   0.970   0.001   0.030
E D  3BP8A                367    0.285  41.112   1.316   0.923   0.002   0.076
E A  3BP8A                368    0.402  44.278   0.657   0.858   0.002   0.139
B M  3BP8A                369    0.219  43.862   0.819   0.694   0.003   0.303
B Y  3BP8A                370    0.159  33.978   0.273   0.502   0.002   0.495
E N  3BP8A                371    0.497  72.775   0.192   0.109   0.005   0.886
E G  3BP8A                372    0.501  39.405   0.016   0.600   0.003   0.397
E S  3BP8A                373    0.531  62.221   0.397   0.782   0.003   0.216
B L  3BP8A                374    0.151  27.593   0.704   0.923   0.002   0.076
B L  3BP8A                375    0.137  25.011   0.416   0.923   0.002   0.076
E I  3BP8A                376    0.474  87.616   0.793   0.923   0.002   0.076
E R  3BP8A                377    0.458 104.974   1.291   0.858   0.002   0.139
B L  3BP8A                378    0.205  37.554   0.719   0.782   0.003   0.216
E L  3BP8A                379    0.294  53.740  -0.643   0.502   0.002   0.495
E Q  3BP8A                380    0.647 115.501   0.651   0.181   0.016   0.803
E G  3BP8A                381    0.871  68.548  -1.101   0.003   0.003   0.994

Explain the output.  Go back.
```

---
